# Professional Growth During and After Completing a Postgraduate Education in Palliative Care – A Qualitative Study

**DOI:** 10.1177/23779608261470480

**Published:** 2026-07-17

**Authors:** Karoline SKEDSMO, Camilla OLAUSSEN, Kristin HOFSØ, Simen A. STEINDAL, Carina LUNDH HAGELIN, Deborah HILDERSON, Andréa Aparecida Gonçalves NES, Dieter SMIS, Hege Vistven STENSETH, Hanne Maria BINGEN

**Affiliations:** 1 155319 Lovisenberg Diaconal University College, Oslo, Norway; 2 Department of Otorhinolaryngology, Head and Neck Surgery, Division of Surgery and Specialized Medicine, Oslo University Hospital, Oslo, Norway; 3 Department of Postoperative and Intensive Care Nursing, Division of Emergencies and Critical Care, Oslo University Hospital, Oslo, Norway; 4 Institute of nursing, Faculty of Health Studies, 87368 VID Specialized University, Oslo, Norway; 5 Department of Healthcare Sciences, Palliative Research Centre, 7643 Marie Cederschiöld University, Stockholm, Sweden; 6 Department of Neurobiology, Care Sciences and Society, Division of Nursing, Karolinska Institutet, Stockholm, Sweden; 7 71325 Centre for Research and Innovation in Care (CRIC), Workforce management, Health Systems and Outcome Research in Care Group, Karel de Grote University College, Antwerpen, Belgium; 8 Department of Caring and Ethics, Faculty of Health Sciences, University of Stavanger, Stavanger, Norway; 9 Department of Mother and Child care, ZAS Hospitals, Antwerp, Belgium

**Keywords:** nursing education, palliative care, active learning, metacognition, critical thinking

## Abstract

**Background:**

The number of patients requiring palliative care is expected to increase in the future. Nurses represent the largest group of healthcare professionals and provide patient care seven days a week. To deliver high-quality palliative care, nurses need education and training.

**Objective:**

This study aimed to explore how newly graduated palliative care nurses experienced and perceived the relevance and impact of their education on professional growth and clinical work during and after completing the postgraduate education.

**Methods:**

An exploratory descriptive design was applied using a qualitative method. Five semi-structured individual interviews were conducted with graduated palliative care nurses after returning to clinical work. Data were analysed using systematic text condensation.

**Results:**

Three categories were identified from the analysis: 1) relevant simulation-based learning to boost learning under and after education, 2) an in-depth understanding of holistic care, and 3) awareness of learning as a transformative process.

**Conclusions:**

Nurses' responsibility in situations where futile treatment is continued near the end of life or during transitions to palliative care underscores the need for nurses to possess metacognitive skills, such as critical thinking, in addition to relevant factual knowledge and technical skills. After pursuing a postgraduate education in palliative care, nurses experience professional growth that continues after graduation. The education led to enhanced attention to, and application of, skills essential for metacognition, stimulating critical thinking and impacting their clinical work. As graduated palliative care nurses, they felt valued as resources by their colleagues.

## Background

The aim of palliative care is to improve the quality of life of patients and their families who are facing problems associated with life-threatening illness (World Health Organization, 2020). Nurses represent the largest group of healthcare professionals and have a wide range of responsibilities and roles in caring for patients and their families in palliative care (Martins Pereira et al., 2021; Moran et al., 2024). In a systematic review, Hökkä et al. (2020a), identified six diverse nursing competencies needed in palliative care; competency to collaborate with the patient, family and the team; competency in communication and cultural issues; clinical competency; ethico-legal competency; psychosocial and spiritual competency; and competency related to a nurse´s professional role and leadership. Nurses need to be prepared through education and training to deliver high-quality palliative care (Martins Pereira et al., 2021), integrating physical, social, psychological and spiritual care in a holistic approach towards patients and their families (Bryk et al., 2023). To ensure adequate and relevant palliative care curricula in education for nurses, recommendations for curricula have been developed, such as in the United States, where the American Association of Colleges of Nursing has made recommendations for implementing palliative care curricula in undergraduate nursing education nationwide (Lippe et al., 2022).

However, graduate nurses report that they are not well prepared to provide palliative care (Malone et al., 2016; Zheng et al., 2016). Studies suggest that nurses lack skills such as having conversations with patients and their families in palliative care and how to take care of the dead body (Croxon et al., 2018; Henoch et al., 2017; Malone et al., 2016; Nilsson & Hommel, 2024). The lack of adequate education and training at the undergraduate level highlights the need for postgraduate education to enhance nurses’ knowledge, critical thinking capabilities and skills (Abu-Qamar et al., 2020; Marciniak et al., 2023). In the health system today, many patients are overtreated in hospitals near the end of life (Sallnow et al., 2022). Nurses need education to develop strong advocacy skills in ethically difficult situations, to take the role as the patient´s guide, liaison and advocate, and provide wise clinical judgement in a complex and rapidly changing environment (Josephsen, 2014; McSteen & Peden-McAlpine, 2006). To prepare nurses to provide palliative care and engage in conversations with patients and their families, education needs to incorporate competencies such as self-reflection, self-awareness and emotional awareness (Hökkä et al., 2024; Stenman et al., 2026). Self-reflection and higher-order thinking skills are essential for metacognition (Josephsen, 2014; Sandvik & Hilli, 2022; Tezer, 2024), and considered as generic skills, highlighted as one key aim of higher education today (Tuononen et al., 2022). Active learning can be defined as ‘a student-centered approach to the construction of knowledge focused on activities and strategies that promote higher-order thinking’ (Doolittle et al., 2023, p. 1), such as role-play, problem-based learning and simulations (Doolittle et al., 2023). Active learning has been recommended in palliative care education to provide students with the necessary experiences and competencies (Hökkä, 2020b; Paal et al., 2019).

### Review of Literature

Elsner et al. (2016) underlined the need for active rather than passive learning methods in palliative care education. They advocated research exploring the most suitable approaches and methodologies for teaching palliative care. A review by Skedsmo, Nes, et al. (2023b) concluded that simulation-based learning in postgraduate nursing educations in palliative care seems to facilitate experiences of personal growth. In a qualitative study of postgraduate palliative care nursing students’ experiences with simulation-based learning, the students expressed the need to be challenged in order to experience professional growth and mastery (Skedsmo, Bingen, et al., 2023a).

Marciniak et al. (2023) assessed and compared the outcomes of postgraduate education and training in palliative care. Assessment forms to evaluate nurses’ and physicians’ learning were provided by the course instructors immediately after the courses. However, the need for evaluation to take place after returning to clinical work in order to assess the clinical relevance of the course and explore the transition from education to clinical work was highlighted (Marciniak et al., 2023). Returning to clinical work and adjusting to a new role may be challenging. Relster et al. (2023) found that after completing their master’s degrees, students experience difficulties when returning to clinical work, regardless of their connections to practice during their education and whether they returned to a previously known or unknown setting. At the undergraduate level, students also experience challenges, and Duchscher (2009) found that the transition from student to newly graduated nurse could be perceived as a shock. The transition from theoretical knowledge in education to the practical application of nursing has been characterised as a gap (Greenway et al., 2019).

A systematic review, synthesising evidence of the implications of postgraduate nursing qualifications on patient and nurse outcomes, found that postgraduate studies could improve knowledge, skills and higher-order thinking, as well as personal views of benefits, such as the perceived enhancement of knowledge and skills. However, out of the 20 studies included, only two involved participants with postgraduate education in palliative care (Abu-Qamar et al., 2020).

### Objective

On the basis of recent literature searches, little is known about newly graduated palliative care nurses' experiences and perspectives of completing a postgraduate education in palliative care after returning to clinical work. According to previous research, returning to clinical work and adjusting to a new role after graduation can be challenging. Consequently, the aim of this study was to explore how newly graduated palliative care nurses experienced and perceived the relevance and impact of their education on professional growth and clinical work during and after completing the postgraduate education.

## Methods

### Design

This study employed a qualitative approach with an exploratory and descriptive design (Hunter et al., 2019) using individual interviews. This design was deemed appropriate because of the lack of previous research on the topic and the aim of describing the experiences of completing a postgraduate education in palliative care from newly graduated palliative care nurses’ perspectives. Semi-structured individual interviews were conducted to provide an opportunity to create an atmosphere of confidentiality, trust and discretion (Polit & Beck, 2021).

### Setting and Sample

The study was conducted at a university college in Norway that offers a postgraduate education in palliative care. In the spring of 2022, 61 registered nurses and two students from other professional backgrounds constituted the cohort of nearly graduated palliative care students. Of these, students who were registered nurses and had attended all mandatory learning activities (n = 61) were invited to participate. They received both written and oral information about the study and were informed that their participation involved attending individual interviews after returning to clinical work as graduated palliative care nurses. Five students agreed to participate, and they signed informed consent forms. One of the researchers (HMB), who was not involved in the course, administered the study invitations.

### Learning Activities in Postgraduate Palliative Care Education

The postgraduate palliative care education is a part-time, one-year education (August–May) that awards 30 European Credit Transfer and Accumulation System credits. The education combines learning on campus with online learning, defined as blended learning (Vallée et al., 2020). To qualify for admission to the education, students must have completed a three-year (180 credits) degree in health or social sciences and possess at least one year of relevant clinical experience. During their studies, students must hold a clinical position relevant to the programme. The postgraduate palliative care education consists of three different courses (see Table 1). The curricula cover the ten core competencies in palliative care as described by the European Association of Palliative Care (EAPC) (Gamondi, Larkin, et al., 2013a; Gamondi, Payne, et al., 2013b), and the six nursing competencies described by Hökkä, Lehto, et al. (2020). There is no clinical placement during the postgraduate education.

**Table 1.** Description of the Courses, Mandatory Learning Activities, Outcomes and Exams

Course	Credits	Mandatory learning activities	Learning outcomes in the mandatory learning activities	Exams
Theoretical foundation	10	Individual written assignment	• Immersing oneself in literature on the hospice philosoph • Reflecting on how the hospice philosophy can be applied in professional practice • Learning to write academic text • Learning to conduct a literature search	Individual written home exam that can be worked on throughout the semester Further development of the written assignment (mandatory learning activity) after feedback from the educator is received
Patient-centred palliative care	15	Individual video-based communication training and reflection Short description of the learning activity: Students acted as nurses in a video-recorded role-play of a communication scenario, which was analysed afterwards.	Exploring communication in challenging situations within palliative care through role-play Reflecting on communication with patients in the palliative phase and with their relatives	Short (4 hours) individual written home exam
		Simulation-based learning Short description of the learning activity: Students acted as nurses in two different scenarios, which were reflected upon in debriefing sessions afterwards. Experienced facilitators, who were not familiar with the students, acted as patients or relatives in the scenarios.	Specified learning outcomes in each scenario Overall learning outcomes in the learning activity: • Practising assessing, evaluating and alleviating complex symptoms and distress • Practising the application of professional knowledge in patient scenarios • Training on communicating about professional issues, analysing and drawing conclusions • Practising effective communication and collaboration with patients or family members, ensuring their involvement and well-being • Sharing experiences and insights into palliative care and clinical challenges	
Service provision for incurable sick and/or dying patients	5	Group project and poster presentation	• Collaborating in groups to explore improvements in service provision and/or the enhancement of service quality • Engaging in an in-depth literature review on a chosen topic and actively incorporating professional experiences • Reflecting on how relevant literature can be applied to improve the quality of palliative care services • Learning to prepare, plan and deliver poster presentations	

### Ethics

The study was approved by the Norwegian Agency for Shared Services in Education and Research (reference number: 115206) and the head of the department at the university college. Participation was voluntary, based on written informed consent and performed in accordance with The Personal Data Act (2018). In education research, there is a possible power imbalance in the student–educator relationship (Leentjens & Levenson, 2013). As the participants for this study were recruited before graduating from the palliative care education, the need to inform all eligible participants of their right to not participate or to withdraw from the study without giving a reason and that it should not affect their further education was emphasised. It was strongly stressed that choosing to participate or not would have no consequences for their educational progression or evaluation. The students were also assured that the course manager (KS) would remain unaware of their participation in the study.

### Data Collection

Five semi-structured individual interviews were conducted with graduated palliative care nurses in October 2022. The semi-structured interview guide (supplementary file 1) included topics such as the impact of the postgraduate education on perceptions of mastery and professional self-esteem and the relevance of active learning methods to clinical work, which enabled the researchers to explore the perspectives and details most significant to graduated palliative care nurses (Polit & Beck, 2021). The semi-structured interview guide was based on previous research (Polit & Beck, 2021). The interviews were conducted and audio recorded using videoconferencing software (Zoom). HMB conducted four of the interviews, while KH conducted the last interview. The researchers who conducted the interviews are both female experienced teachers and interviewers, not involved in the course or postgraduate education. KH is a critical care nurse and professor, while HMB is a senior educational advisor. The interviews lasted from 24 to 45 minutes, with an average of 30 minutes. These were carried out five months after the last exam and six months after the last mandatory learning activity. This timeframe facilitated a situation in which the participants had time to experience the transition from being part-time students to graduated palliative care nurses and reflect upon their experiences (Duchscher, 2009).

### Analysis

**Figure 1.**
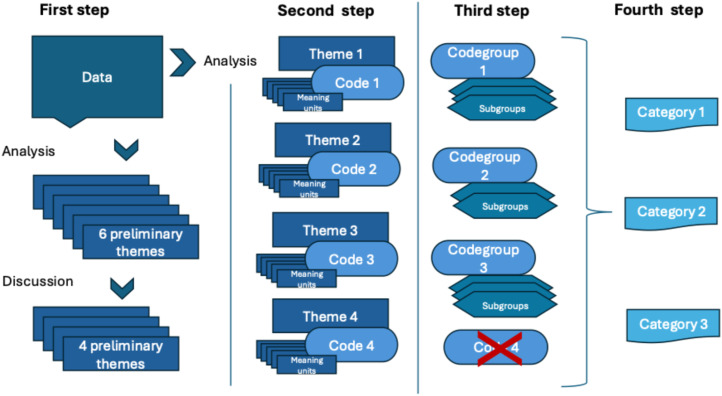
Illustration of the steps in the analytical process

The study was reported according to the Consolidated Criteria for Reporting Qualitative Research (Tong et al., 2007). The interviews were transcribed verbatim by an external transcriber. The transcribed interviews were analysed using systematic text condensation (Malterud, 2012). NVivo (Alfasoft, Norway) was used to store and organise the material in the first three steps of the analysis, supported by tables in Microsoft Word. First, the interviews were read several times to gain an impression of each interview and of the whole data material. Guided by the aim of the study, six preliminary themes were identified. Through discussion, these preliminary themes were collapsed into four preliminary themes. In the second step, meaning units related to the preliminary themes were identified and sorted, marking them with codes. The preliminary themes were then developed into code groups, and meaning units were sorted into four code groups. In the third step, the meaning units within each code group were arranged into subgroups, with four code groups reduced to three code groups and eight subgroups. The meaning units within each subgroup were reduced into a condensate. Fourth, the content under each code group was summarised into an analytical text presented in three categories. The analytical text presents the most salient content and meaning. In the first step of the analytical process KS, CO and HMB (all female) all read the data from the interviews separately and then suggested themes. In the next three steps of the analytical process, KS did the first part of the procedure and presented a proposal that was discussed with CO and HMB and then revised after discussion. An illustration of the steps in the analytical process is presented in Figure 1, while an example of the analytical process is given in Table 2. All authors agreed upon the final categories. Transcripts and results were not returned to the participants for member checking.

**Table 2.** Analytical Process

Impression of the whole	Identifying and sorting meaning units – from themes to codes		Condensation – from code to meaning		Synthesising
Preliminary theme	Code group	Meaning units (selected)	Subgroup	Condensate (selected part)	Category
Learning as the acquisition of measurable knowledge and skills versus learning as transformational	An inner transformative process	*‘The education made me feel like I’m standing steadier on my feet when it comes to palliative care. But at the same time, I can’t put my finger on what I know more now compared to what I knew when I was just starting. But I think that there must be something’. –* Nurse 2 *‘You initiate a reflection process that enables you to see yourself perhaps a little more from the outside. Hmm’ –* Nurse 1	The education facilitating transformation to become a more reflective nurse	The education in general has made me feel like I’m standing steadier on my feet when it comes to palliative care. But at the same time, I can’t put my finger on what I know more now compared to what I knew when I was just starting. But I think that there must be something. Yes, I guess it’s just that I feel that I stand stronger and more secure. [The active learning activities] start a reflection process in which you see yourself from the outside.	Awareness of learning as a transformative process

## Results

The participants had varied backgrounds as nurses with experience in both primary health care and hospitals. One of the participants had experience working in specialist palliative care, while another had additional postgraduate nursing education. The participant descriptions are provided in Table 3.

**Table 3.** Description of the Participants (n=5)

Gender	​
Female	5
Age, median (range)	44 (33–56)
Experience as registered nurse, median (range), years	9 (3–27)
Experience in palliative care, median (range), years	11 (3–15)

Three categories were identified from the analysis: 1) Relevant simulation-based learning to boost learning under and after education, 2) An in-depth understanding of holistic care, and 3) Awareness of learning as a transformative process (Table 4).

**Table 4.** Presentation of Categories and Subgroups

Categories	Subgroups
Relevant simulation-based learning to boost learning under and after education	Experience practical application of theoretical knowledge
	Challenging learning situations providing confidence in practice
	Communication as an inherent skill or a skill that can be developed
An in-depth understanding of holistic care	A broad assessment of the patient, which is conveyed to peers
	The patient’s relatives as a part of the extended clinical gaze
Awareness of learning as a transformative process	The education facilitating the nurses to become more reflective
	Perceptions of acquired professional confidence
	Being perceived as a professional resource in palliative care

### Relevant Simulation-Based Learning to Boost Learning Under and After Education

Simulation-based learning was highlighted among the participants as a learning activity in the postgraduate education that had enhanced their confidence in palliative care. Its importance for boosting confidence was articulated in two distinct ways. First, by confronting challenging clinical situations through relevant simulated scenarios, the participants gained a sense of familiarity and sense of mastery towards similar clinical situations afterwards. Second, enhanced confidence was achieved by receiving constructive and affirmative feedback from their peers on their own performance. One participant had feared causing burden to patients and their families because of the participant’s own communication style but because of feedback from peers, this participant learned to become more confident in navigating the delicate balance between being honest and considerate in challenging conversations. During and after their participation in the simulation-based learning activities, the participants also had the experience of integrating theory and applying it into practical situations, thus increasing the quality of their clinical work: *‘The scenario was with a [realistic] patient we could meet at work, so the scenario was okay. It was very useful and effective. Although the situation wasn’t real, I perceived it as very realistic when I participated. I treated the actor like a patient whom I should do my best for and try to use the things we’ve learned—the knowledge—in a realistic situation. Then, you transfer it into practice in an effective way’. –* Nurse 5

Most participants regarded communication as both a cornerstone of palliative care and the domain most prone to error. They suggested adding more simulation-based learning activities to enhance future students’ personal growth and confidence in palliative care, as such activities had helped refine their communication skills and increased their awareness of their capacity to use communication effectively in managing conversations with patients and their families. To support patients in making informed decisions and ensuring their right to self-determination, some participants emphasised the need to practice initiating challenging patient conversations, especially at an early stage in the patient’s disease trajectory, and to incorporate more targeted simulated scenarios that focus on communication. Conversely, others felt that they were already trained communicators because of their experiences working as professionals and, therefore, desired opportunities to practise technical skills and procedures. However, in the simulation activities that they had attended as palliative care students, they had seen a potential for their own improvement in this domain by observing and learning from peers whom they perceived as naturally adept communicators: *‘Some people are, in a way, born to communicate. They just do it automatically without thinking about it. So, it’s really nice to hear that they have actually reflected on it along the way and have consciously chosen a strategy, right? That it’s actually possible to kind of take with you those sentences, those words, that they’ve used [for your own use]’. –* Nurse 3

Clinical palliative care situations that were perceived as challenging, such as conversations with children about their parents or siblings who were in a palliative state or dying, were suggested as possible starting points for additional simulated scenarios. Opportunities to practise simulated conversations with children could lower the threshold and reduce avoidance of having these conversations in real-life practice: *‘I think it’s a good idea to practise because health personnel avoid these conversations. Maybe that’s the thing that health personnel avoid the most. Relatives are okay, but children as relatives, that’s scary. Because you’re afraid to cause more burden. Then, you avoid them, and they lose the opportunity to be seen and met. And if you don’t start to have those conversations, you’ll never have experience either’. –* Nurse 5

### An In-Depth Understanding of Holistic Care

During their postgraduate education, the participants became more aware of the importance of conducting holistic assessments of patients, emphasising not only a patient’s physical but also psychological, social and spiritual needs. This awareness significantly altered their current clinical practices in evaluating the needs and circumstances of patients requiring palliative care. The participants emphasised making patient-centred plans for future care before a patient’s condition worsens. A participant described this as follows:*‘When a patient is in the emergency or critical care units, we do all we can, it’s all in, right? It´s not always doing what’s best for the patient in a palliative state, we go a little hard and focus on cure. But it’s a lot we cannot cure. I’ve been more aware of that. That the patients have quite different needs.’ -* Nurse 3

The participants also communicated their assessments of patients differently to their multidisciplinary colleagues after completing postgraduate education, with a holistic approach to the patients’ situations and a goal of communicating and reaching consensus in the best interests of the patients. After completing postgraduate education, the participants became highly aware of the need to share information and engage in conversations with relatives. They described having evolved in how they perceived, communicated with, and included the patients’ relatives in various situations. Participants provided examples of how they explore the relatives’ understanding of the situation after attending a meeting with the patient’s doctor or when observing that the patient’s condition is worsening. Furthermore, the participants described being more focused on ‘tuning in’ to where the relatives were: *‘I focus more on listening and being calm. I’ve been that nurse who always speaks. But now, I’ve learned to make it short and clear and try to listen to what the relatives have to say before I tell them my opinion. I’ve become more service oriented; I’m telling them that I’m here if they need me. And they should just call on me or get to the hallway. I’m not walking straight into a situation before I get a sense if the relatives want me to be there’. –* Nurse 4.

### Awareness of Learning as a Transformative Process

The participants described completion of the postgraduate education in palliative care as a comprehensive transformation process which persisted beyond the year of their education and fostered transformative shifts in their own clinical work. They experienced becoming more reflective and confident, applying critical thinking as an integrated part of their clinical work. Active learning activities containing initiated reflections were highlighted as learning activities and processes that particularly contributed to their transformation. Through such activities, participants were able to see themselves from the outside, discern their own performances objectively, and thereby gain heightened awareness of their own competencies and areas for improvement. Despite these examples, some participants found it challenging to verbalise or pinpoint specific examples of new knowledge or competencies that they had obtained: *‘The education made me feel like I’m standing steadier on my feet when it comes to palliative care. But at the same time, I can’t put my finger on what I know more now compared to what I knew when I was just starting. But I think that there must be something’. –* Nurse 2

After completion of the postgraduate education participants described their current professionality as founded in knowledge and confidence because of their postgraduate education in three areas that they actively applied as palliative care nurses: 1) reasoning with colleagues, 2) observing patients and 3) assessing situations. Knowledge and confidence were regarded as an important foundation when they had to reason for what they believed was professionally right for their patients. Facilitating colleagues’ awareness of recognising patients potentially needing palliative care to enable them to make appropriate decisions and adopt an appropriate approach towards these patients’ treatments, was highlighted as important among the participants. Additionally, the participants highlighted the importance of having confidence in observing patients and assessing their situations. They also perceived themselves as being more aware of situations in which they were continuing with futile treatment towards the end of a patient’s life. This awareness made them initiate collective reflections with colleagues about the hesitancy to dare to intervene in situations in which a patient’s death was imminent. The professional confidence that the participants gained during their postgraduate education was perceived as a resource under challenging situations: *‘Because it [the postgraduate education in palliative care] probably did something to me. I’m actually more confident at work, and I don’t fear going into difficult conversations, clinical rounds or anything. I feel that I’m more confident in the situation, and the situations are not so scary anymore ....’–* Nurse 1

The participants also described the postgraduate education as transformation process that enabled them to take on the roles of mentors and facilitators of reflection among their colleagues in clinical settings. The participants described how their current colleagues perceived them as professional resources in palliative care, either when these colleagues needed supervision themselves or when undergraduate nursing students in clinical placement required supervision. They reflected on the importance of their roles as palliative care resices and the need to manage these roles wisely. A challenge that participants encountered after completing the education was that their colleagues expected them to take responsibility for all patients in need of palliative care: *‘Many of my colleagues suggest that I can take over [the patient] because I have a postgraduate education in palliative care. But I find it important to teach them and guide them to be confident in the situation’. –* Nurse 4

## Discussion

The main findings of this study indicate that the graduated palliative care nurses experienced postgraduate education as a transformative process towards becoming critical reflective and confident nurses. This process persisted after graduation and made an impact on their clinical work. Aligned with findings in this study, experiences of education in palliative care as a transformative process have been described among undergraduate nursing students (Melin-Johansson et al., 2018). However, previous postgraduate studies exploring experiences after returning to work are limited (Abu-Qamar et al., 2020; Marciniak et al., 2023), and the findings from this study extend prior work as interviews were conducted after graduation and upon returning to clinical work.

Researchers argue that education should focus on metacognitive skills rather than factual knowledge and technical skills (Josephsen, 2014; Sandvik & Hilli, 2022; Tezer, 2024). Generic skills like metacognition, are essential in the twenty-first century (Tuononen et al., 2022). When reflecting on the outcomes of their postgraduate palliative care education, the participants illustrated how reflection throughout their education and learning activities facilitated a continuing transformative process, emphasising enhanced awareness and critical thinking. This aligns with the theory of transformative learning by Mezirow (1997), in which the adult learner, through education, reframes earlier assumptions and expectations, leading to changes in their ways of thinking, acting, and feeling, and in their frame of reference. Frames of reference are the structures of assumptions through which adults understand their experiences (Mezirow, 1997). Critical reflection is an important process for facilitating transformative learning and changes in the frames of reference (Mezirow, 1997; Van Schalkwyk et al., 2019). In the present study, participants described how experiences and activities during postgraduate education significantly altered their understanding of clinical practice and led them to apply critical reflection as an integral part of their clinical work.

The participants had varying perspectives on what could be learned and what they considered innate abilities. Some questioned whether learning and practising nontechnical skills were necessary or whether the focus should be more on technical skills and procedures during their education. Technical skills may be perceived as more accessible educational outcomes until a maturation process and change have commenced within individuals themselves through and in the context of their own clinical practice. According to Mezirow (1997) adult learners often have an immediate focus on practical short-term objectives. It is crucial to also recognize and address long-term goals. To promote metacognition, educators must guide students toward establishing a learning environment where students are explicitly engaged in metacognitive activities, where they can ask questions and where they are encouraged to reflect on their thought processes individually and together with peers (Leat & Lin, 2003; Tezer, 2024). Findings in this study suggest that while educators may have effectively integrated skills important for metacognition and long-term goals into students’ palliative care education, they might not have been successful in elucidating metacognitive skills through a significant, deliberate and intended approach.

The participants in the present study focused on what they saw as learnable. During their postgraduate education, students must pass mandatory learning activities and two exams. The focus on these mandatory activities, measurable exam results and measurable skills could overshadow the transformation these students experience through their studies. However, it was after graduating that the participants became aware of this ongoing change in themselves in contact with their colleagues, patients and patients’ relatives. The professional self is an essential component (Ward-Griffin et al., 2012) and an important instrument in palliative care nursing; consequently, practising and developing the professional self through a transformative process are essential (Timmerman & Baart, 2022). Educators can create learning situations in which students can experience limitations, challenges and conflicts and thereby increasingly take responsibility for their own actions and impact on themselves and others (Timmerman & Baart, 2022). The participants’ experiences of postgraduate education as a transformative process align with the findings of Melin-Johansson et al. (2018) from undergraduate clinical placements in palliative care, even though this study´s blended learning design did not include clinical placement. Active learning methods, such as simulation-based learning in postgraduate palliative care education, can provide students with authentic situations in which transformative learning and development of metacognitive skills can occur (Tezer, 2024; Van Schalkwyk et al., 2019). Active learning approaches and reflective exercises during postgraduate education in palliative care facilitate participants’ ability to see themselves from the outside and objectively assess their own performance, as well as heighten their awareness of their competencies and areas for improvement. In the review by Van Schalkwyk et al. (2019), opportunities for reflection in education are highlighted as important for both facilitating and assessing transformative learning. The increased awareness, reflectiveness and continuing developmental process could be seen as a transformative learning approach in which the professional self is changed, strengthened and further developed through and after graduation.

The participants in the present study described an enhanced awareness of their patients’ situations, requiring them to take a step back and assess them from a new perspective. Additionally, the participants described how they used their professional knowledge and confidence when they had to reason with colleagues about what they believed was the right decision for their patients. They described becoming more aware of and taking responsibility for situations in which they were continuing futile treatment near the end of a patient’s life, initiating collective reflections with colleagues. Futile treatment and overtreatment at this stage of life represent a challenge in the 21st century (Sallnow et al., 2022). An important nursing role is to advocate for the patient, guided by wise clinical judgment (Josephsen, 2014; McSteen & Peden-McAlpine, 2006). However, the transition to palliative care represents one of the most challenging situations for nurses to step into as advocates (McSteen & Peden-McAlpine, 2006). Furthermore, previous research has described nurses feeling unprepared for these situations after graduation (Croxon et al., 2018; Henoch et al., 2017; Nilsson & Hommel, 2024). Findings in this study indicate that completing a postgraduate education in palliative care may support nurses in cultivating metacognitive skills, including the regular use of reflection, critical thinking and questioning in daily practice. Cultivating these metacognitive skills may enable nurses to provide sound clinical judgement and engage in patient advocacy within multidisciplinary teams (Josephsen, 2014), as well as prepare them for the requirements of lifelong learning (Davis et al., 2014).

## Strengths and Limitations

In the analytical process, researcher triangulation was applied to enhance the intersubjectivity and credibility of the findings (Polit & Beck, 2021). During each step of the study, KS analysed the data, while CO and HMB asked critical questions and made suggestions. Multiple researchers included in the analytical process enhance confirmability (Korstjens & Moser, 2018). HMB conducted four of the interviews, while KS and CO was not involved in conducting any of the interviews. CO and HMB are not involved in the postgraduate education in palliative care and could ask questions and provide nuances that KS might potentially overlook due to the close connection to the educational programme, minimizing the risk of analytical bias (Smith & Noble, 2025). In addition, questions were asked during the interviews to validate immediate interpretations, and reflexive notes were written during the research process. The identified categories from the analysis were checked against the transcripts (Malterud, 2012), to ensure that findings and interpretations reflect the views of the participants. To ensure dependability, the research process has been described in detail. This allows the reader to evaluate transferability to other contexts and situations (Ahmed, 2024). To ensure rigour and trustworthiness, thick descriptions of the context and participants are provided (Polit & Beck, 2021). The study was reported according to the Consolidated Criteria for Reporting Qualitative Research (Tong et al., 2007) to enhance transparency and dependability.

Potential limitations include self-selection bias, as participants who felt positively about the education may have been more inclined to participate and may have had different experiences than those who didn´t (Hiratsuka, 2025; Smith & Noble, 2025). There is also a risk of social desirability bias, in which the participants provide answers they believe are socially acceptable during the interviews (Bispo Júnior, 2022). Even so, the participants openly shared their experiences and recommendations on what could have been changed in the educational programme. The participant group was smaller than initially planned, but following Braun and Clarke (2025) recommendation, we considered the quality of data to address the aim of the study, rather than the number of participants. The authors considered the number of interviews sufficient to provide rich data on the topic, guided by the concept of information power, taking into account the aim of the study, the sample specificity, established theories, the quality of dialogue in the interviews, and the strategy of analysis (Malterud et al., 2016). The researchers deemed the data in this study sufficient to address the aim, as the aim of the study was narrow (Malterud et al., 2016). The variations in the participants' demographic data and experiences, combined with their specific knowledge related to the study aim, provided a rich and nuanced account of the phenomenon (Hennink & Kaiser, 2022; Malterud et al., 2016; Roberts et al., 2026). The participants had backgrounds and experiences similar to the broader cohort of non-participants. The interviews lasted between 24 and 45 minutes. Considering the quality of the dialogue (Malterud et al., 2016), which was strong and focused, the data material was considered sufficient. The study was planned, and the findings were explained and discussed in relation to established theory, as suggested by Malterud et al. (2016). The interviews were conducted five months after the last exam, allowing participants to experience the transition from part-time students to graduated, working palliative care nurses and to reflect on their practice. However, this timeframe may also have introduced recall bias (Smith & Noble, 2025), and several participants struggled to recall details from their postgraduate education and to distinguish between specific learning activities. Nevertheless, during the interviews, participants were able to elaborate on their experiences of the postgraduate education and described a clear distinction between before and after completing it.

## Implications for Practice

To facilitate the development of skills important for nurses' metacognition and transformation, educators must prepare learning activities that include reflection and awareness, clarifying the value of this type of educational approach. Applying active learning methods that integrate the use of metacognitive skills, such as awareness and reflection, enhances critical thinking in nurses clinical work after graduation. The ongoing maturation process that continues after returning to clinical practice should be incorporated into education to better prepare nurses for this transition.

## Conclusions

Nurses' responsibility in situations where futile treatment is continued near the end of life or during transitions to palliative care underscores the need for nurses to possess metacognitive skills, such as critical thinking, in addition to relevant factual knowledge and technical skills. As nurses feel unprepared for palliative and end of life care after graduation, postgraduate education in palliative care is important to become confident in such situations. After pursuing a postgraduate education in palliative care, newly graduated nurses experience professional growth and enhanced professional confidence. This process continues after graduation, and participants perceived themselves as changed. The education has led to enhanced attention to and the application of skills essential for metacognition, such as awareness and reflection, stimulating critical thinking and having an impact in their clinical work, experiencing themselves as more confident and reflective. This enables the nurses to act as the patients' advocate, take a step back and assess the patients from a broad perspective applying a holistic approach. As graduated palliative care nurses, they experienced being seen as valuable resources in palliative care by their colleagues.

## Supplemental Material

Supplemental Material - Professional Growth During and After Completing a Postgraduate Education in Palliative Care – A Qualitative Study Supplemental Material for Professional Growth During and After Completing a Postgraduate Education in Palliative Care – A Qualitative Study by Karoline SKEDSMO, Camilla OLAUSSEN, Kristin HOFSØ, Simen A. STEINDAL, Carina LUNDH HAGELIN, Deborah HILDERSON, Andréa Aparecida Gonçalves NES, Dieter SMIS, Hege Vistven STENSETH, Hanne Maria BINGEN in Sage Open Nursing
