# Supplementary material for: Professional Growth During and After Completing a Postgraduate Education in Palliative Care – A Qualitative Study
Source: SAGE Open Nurs. 2026 Jul 17;12:23779608261470480. doi: 10.1177/23779608261470480 (PMC13379645; doi:10.1177/23779608261470480)
Supplement: Supplemental Material - Professional Growth During and After Completing a Postgraduate Education in Palliative Care – A Qualitative Study [file sj-pdf-1-son-10.1177_23779608261470480.pdf]

Interview guide for individual interviews

**Categories**	**Main questions**
**Opening/introduction**	Can you tell me about your experiences with postgraduate education in palliative care?
**Postgraduate education influence on experience of mastery and professional confidence**	Can you describe situations where you experienced learning during postgraduate education?
	Have you participated in simulation-based learning or other learning experiences after graduation? Please describe it.
	Can you describe if and how postgraduate education has affected your nursing practice? Please describe it.
	Can you describe how you perceive yourself in interactions with patients and their relatives in clinical practice as a nurse with postgraduate education in palliative care?
**Percieved relevance of postgraduate education in clinical practice**	Can you describe how you have experienced the relevance of postgraduate education for your clinical practice now, after a few months?
	What could have been changed to make postgraduate education more relevant to your clinical practice?
	Can you describe situations in which you have applied what you learned from simulation-based training or other learning activities when interacting with patients and their families?
**Ending/summary**	Is there anything we haven´t covered in the interview that you would like to mention?
